# Low-Temperature xTB–MD–DFT Screening of Functionalized Oxide Surface-Patch Models (TiO_2_, ZnO, CeO_2_) for Hydrocarbon Association and Microbial-Proxy Perturbation Assessment in Cold Bioremediation

**DOI:** 10.3390/nano16130815

**Published:** 2026-07-01

**Authors:** Julio Guerra, Johana Zuñiga, Miguel Gualoto, Tania Oña, Marcelo Cevallos

**Affiliations:** 1 Facultad de Ingeniería en Ciencias Aplicadas, Universidad Técnica del Norte, Ibarra 100101, Ecuador; 2 Centro de Investigación Biomédica (CENBIO), Facultad de Ciencias de la Salud Eugenio Espejo, Universidad UTE, Quito 170527, Ecuador; 3 Facultad de Ingeniería en Ciencias Agropecuarias y Ambientales, Universidad Técnica del Norte, Ibarra 100101, Ecuador; magualoto@utn.edu.ec (M.G.); mcevallos@utn.edu.ec (M.C.)

**Keywords:** cold-environment bioremediation, oxide surface patches, TiO_2_, ZnO, CeO_2_, hydrocarbon association, microbial-interface proxies, xTB, ORCA, molecular dynamics, interfacial selectivity, computational screening

## Abstract

Hydrocarbon biodegradation in cold environments is constrained not only by microbial catabolic capacity but also by interfacial access to poorly soluble substrates and by the way remediation materials interact with microbial envelope-related structures. This study presents an uncertainty-aware low-temperature computational screening workflow for prioritizing functionalized oxide surface-patch models that may favor hydrocarbon association while avoiding excessive perturbation of simplified microbial-interface proxies. Twelve finite oxide–ligand candidates derived from TiO_2_, ZnO, and CeO_2_ patches functionalized with bare, catechol, glycerol, or citric acid states were evaluated against three hydrocarbon probes, hexane, toluene, and naphthalene, and two microbial-interface proxies. The workflow combined GFN2-xTB geometry optimization and relative interaction-energy screening, clean GFN2-xTB/ALPB rescoring with rescue tracking, short xTB-MD perturbation analysis, ORCA refinement of selected candidates, sensitivity analysis of ranking parameters, and integrated evidence classification. The analysis supports interfacial selectivity, rather than maximum adsorption strength, as the central design principle. TiO_2_–catechol and TiO_2_–glycerol remain experimentally testable primary candidates because their original screening profile combines chemically interpretable hydrocarbon association with comparatively mild microbial-proxy interaction descriptors. ZnO–catechol and ZnO–glycerol emerged as sensitivity-competitive secondary candidates under several scoring assumptions. Completed short xTB-MD trajectories further showed that TiO_2_–glycerol produced moderate perturbation against the peptide proxy, whereas TiO_2_–glycerol against NAG and ZnO–catechol against the peptide proxy showed very high proxy displacement. Overall, the workflow provides a transparent prioritization framework for experimental validation.

## 1. Introduction

Petroleum hydrocarbons remain persistent in cold environments because temperature controls mass transfer, microbial activity, and substrate turnover. Arctic soil microcosms show slower hydrocarbon loss under low-temperature and freeze–thaw conditions [1]. Cold-adapted actinobacterial strains still degrade n-alkanes, phenol, anthracene, and pyrene at 15 °C and below [2]. Specialized hydrocarbonoclastic bacteria are also well documented in cold systems, including *Oleispira antarctica* from Antarctic coastal seawater [3], Arctic crude-oil-degrading consortia [4], and psychrotolerant *Rhodococcus* strains from polluted Antarctic soils [5]. These studies define a strong biological foundation for low-temperature hydrocarbon bioremediation, while they also show that cold adaptation alone does not fully resolve the remediation bottleneck.

That bottleneck is largely interfacial. Hydrocarbon biodegradation depends on how efficiently microorganisms access poorly soluble substrates, and bioavailability strongly regulates degradation rates in contaminated soils. Directed movement toward aromatic hydrocarbons can further improve substrate encounter and degradation efficiency, as shown for naphthalene-degrading bacteria in heterogeneous aqueous systems [6]. In practical terms, the rate-limiting step often shifts from metabolic capability to the physical and chemical conditions that govern contact among the hydrocarbon phase, the microbial envelope, and the surrounding aqueous medium.

Nanomaterials offer a plausible route to manipulate this interfacial bottleneck. Oxide nanoparticles provide high surface area, tunable surface chemistry, and strong adsorption capacity for hydrophobic molecules, and they can accelerate crude-oil biodegradation when combined with bacterial degraders, as reported for Fe_2_O_3_ and ZnO nanoparticles [7]. Metal oxide nanoparticles also interact directly with bacterial surfaces and membrane-associated structures, and these interactions depend on surface composition, charge, and nanoscale geometry [8]. At the bacterial-envelope level, nanoparticle association is strongly governed by lipopolysaccharide density and structure in Gram-negative outer membranes [9], and oxide nanoparticles interact measurably with bacterial cell-envelope biomolecules, including membrane- and wall-associated components [10]. These reports position adsorption promotion and microbial-proxy interaction profile as two coupled design variables in nano-assisted bioremediation [11].

The present study addresses this coupled design problem through a reduced molecular screening approach. Rather than modeling complete nanoparticles, aggregated colloids, protein coronas, explicit hydration shells, or complete bacterial envelopes, the work focuses on finite oxide surface-patch models and chemically simplified microbial-interface proxies. This scope allows local oxide–ligand interfacial behavior to be compared under a consistent computational protocol. Accordingly, the central question is which oxide–ligand interface within the evaluated candidate space provides the most defensible compromise between hydrocarbon association and low microbial-proxy perturbation under low-temperature screening conditions.

The contribution of this work is therefore an uncertainty-aware interfacial prioritization framework. The framework integrates relative hydrocarbon-association descriptors, microbial-proxy interaction descriptors, structural perturbation indicators, DFT refinement when available, sensitivity analysis, and explicit uncertainty flags for extreme semiempirical descriptors and nonconvergent refinement cases. The working hypothesis is that oxide surfaces with an intermediate association regime and a chemically moderated ligand shell may provide a more useful interface than surfaces that simply maximize raw hydrocarbon affinity. Within that hypothesis, TiO_2_, ZnO, and CeO_2_ finite surface patches functionalized with glycerol, catechol, or citric acid provide chemically distinct but comparable platforms for testing how oxide identity and ligand chemistry influence the adsorption–proxy-perturbation balance.

The objective of this study is to rank candidate interfaces through a low-temperature xTB–MD–DFT workflow and to classify them as primary experimental candidates, sensitivity-competitive secondary candidates, warning cases, or low-priority cases. The resulting ranking is intended as a hypothesis-generating prioritization tool for experimental validation rather than as a definitive prediction of whole-nanoparticle behavior, microbial toxicity, or biodegradation performance.

### 1.1. Theoretical Framework

#### 1.1.1. Low-Temperature Hydrocarbon Biodegradation as an Interfacial Problem

Hydrocarbon biodegradation in cold environments depends on the coexistence of two conditions: microbial catabolic capacity and efficient contact with poorly soluble substrates. Arctic soil studies show that psychrotolerant microorganisms can mineralize hydrocarbons at low temperature, including dodecane at 7 °C, and that nutrient supply and physicochemical soil properties strongly regulate the biodegradation response [1,12]. Broader cold-environment literature also shows that hydrocarbon turnover remains feasible across alpine, polar, and other extreme systems when adapted microorganisms are present [13,14]. This body of work defines cold hydrocarbon biodegradation as an active process with clear kinetic constraints rather than as a purely metabolic limitation.

The microbial component of this process is well established. Obligate or strong hydrocarbon degraders have been described in cold marine and polar systems, including *Oleispira antarctica* as a psychrophilic hydrocarbon degrader from Antarctic coastal seawater [3], its later genome-level functional characterization [15], and psychrotolerant *Rhodococcus* strains from polluted Antarctic soils [5]. These organisms support the premise that hydrocarbon catabolism at low temperature is biologically realistic. The remaining challenge lies in how efficiently the microbial cell can reach and engage the hydrocarbon phase.

That challenge is governed by bioavailability. The relation between hydrocarbon biodegradation and fuel-oil bioavailability in soil has been demonstrated directly, with accessible contaminant fractions controlling biodegradation rates. In parallel, bacterial chemotaxis toward naphthalene has been shown to enhance degradation in heterogeneous aqueous systems [6]. Together, these studies establish that the decisive variable is often the interface among the microbial surface, the hydrocarbon phase, and the aqueous medium. This theoretical point directly supports a screening strategy centered on nanoparticle-mediated interfacial tuning.

#### 1.1.2. Oxide Nanoparticle Surfaces as Tunable Adsorption-Active Interfaces

Metal oxide nanoparticles provide a relevant platform for interfacial engineering because they combine a high surface-to-volume ratio, chemically addressable surface sites, and strong adsorption capability. In remediation-oriented studies, oxide nanoparticles have already been shown to accelerate crude-oil biodegradation when combined with degrading bacteria, including the use of ZnO and Fe_2_O_3_ nanoparticles in mixed bacterial systems [7]. At a broader level, inorganic nanoparticles are recognized as chemically versatile systems whose physicochemical properties can be engineered to control biological interactions [16]. This framework places oxide nanoparticles in a dual role: adsorption-active materials and surface-defined biological interfaces.

The present study focuses on TiO_2_, ZnO, and CeO_2_ because these oxides represent chemically distinct yet tractable oxide surface-patch families. TiO_2_ and ZnO are widely recognized as oxide nanomaterials with pronounced biological activity at interfaces, including direct interaction with bacterial-envelope biomolecules [10]. More generally, bacterial responses to metal and metal oxide nanoparticles are strongly influenced by material identity, surface chemistry, and local interfacial structure [17,18]. These observations support the selection of oxide composition as a primary design variable in the present work.

Surface functionalization is the second primary variable. Surface functionality governs how nanoparticles interact with biological interfaces, including adhesion, uptake, and membrane contact behavior [19,20]. Catechol is especially relevant because it binds strongly to titanium oxide surfaces and forms robust interfacial coordination states, as shown by DFT and spectroscopy on TiO_2_ surfaces [21,22]. Citric acid is also a well-established oxide-surface stabilizer and electrosteric coating ligand in nanoparticle systems [23]. In this framework, catechol represents a strong anchoring aromatic ligand, citric acid represents a multicarboxylate hydrophilic ligand, and glycerol represents a hydroxyl-rich polyol surface state. These ligand classes are therefore appropriate for probing how surface chemistry modulates the balance between hydrocarbon association and microbial-proxy interaction profile.

#### 1.1.3. The Microbial Envelope as the Critical Proxy Target

The nanoparticle–microorganism interface is defined by the bacterial cell envelope. In Gram-negative bacteria, the outer leaflet of the outer membrane is enriched in lipopolysaccharide (LPS), a tripartite amphiphile composed of lipid A, a core oligosaccharide, and an O-antigen polysaccharide chain [24]. In parallel, peptidoglycan is the structural polymer that protects bacteria from osmotic rupture and maintains cell shape across bacterial envelopes [25,26]. These two envelope components define the main physical and chemical barriers that any nanoparticle-assisted hydrocarbon strategy must accommodate.

Nanoparticle interaction with this envelope is already known to depend strongly on envelope composition. The extent and distance of nanoparticle association with Gram-negative outer membranes are governed by LPS density and structure [9]. Oxide nanoparticles also induce measurable changes in bacterial-envelope biomolecules, including shifts associated with proteins, phospholipids, peptidoglycan-related components, and LPS-rich surfaces [10]. More recent theoretical and simulation-focused work has further established that the bacterial cell envelope must be treated as a multilayered, chemically heterogeneous barrier rather than as a single generic membrane [27]. These findings justify the use of microbial-envelope proxies in the present study and support the hypothesis that microbial-proxy perturbation must be evaluated directly at the interface level.

Within that framework, the microbial-interface proxies used here should be interpreted as reduced chemical representatives of envelope-related motifs, not as full cell-envelope models. The NAG-based proxy approximates a carbohydrate-rich motif relevant to peptidoglycan- and polysaccharide-associated interfaces, whereas the GGSTNQ peptide proxy represents an exposed polar peptide fragment that can report nonspecific interaction with protein-like or peptidoglycan-associated surface chemistry. These proxies are most relevant to Gram-negative hydrocarbon-degrading bacteria because the outer membrane and periplasmic envelope include lipopolysaccharide-rich, polysaccharide-rich, peptidoglycan-associated, and protein-containing regions. Oleispira antarctica is used in the manuscript as an example of a cold-adapted hydrocarbonoclastic Gram-negative bacterium rather than as the only possible biological target.

#### 1.1.4. Molecular Simulation Framework

The central hypothesis of the present study is interfacial and multiscale. It links adsorption, microbial proxy interaction profile, and low-temperature behavior. This type of hypothesis is well-suited to a tiered molecular modeling strategy. Semiempirical quantum methods provide the throughput needed to compare many candidates and poses, while molecular dynamics resolves the structural response of the microbial proxy to candidate binding, and DFT refinement improves the energetic resolution of the top candidates. This logic matches the current direction of cell-envelope and nanoparticle-interface research, where molecular simulation is used to resolve how local surface chemistry controls membrane association, barrier traversal, and envelope perturbation [26,27].

In this theoretical context, the adsorption problem is defined by the energy of nanoparticle–hydrocarbon association, whereas the microbial-proxy perturbation problem is defined by the energy and structural consequences of nanoparticle interaction with bacterial-envelope proxies. The most favorable candidate is therefore expected to combine a sufficiently strong hydrocarbon-binding profile with a mild envelope-perturbation profile. This dual criterion directly supports the study hypothesis and provides the conceptual basis for the integrated MD–DFT screening strategy developed in the present work.

## 2. Materials and Methods

### 2.1. Computational Workflow, Revision Environment, and Uncertainty-Tracking Logic

The study used a reproducible Python workflow implemented in the custom pipeline npbio_screen. The original screening layer included structure preparation, GFN2-xTB geometry optimization and relative interaction-energy screening, short low-temperature xTB-MD perturbation analysis, ORCA refinement of selected shortlisted candidates, and integrated multi-criteria ranking. The workflow added four explicit uncertainty-tracking layers: (i) clean GFN2-xTB/ALPB single-point rescoring of all candidate, analyte, and complex structures using a consistent energy protocol; (ii) Tier-1 and Tier-2 rescue calculations for initially nonconvergent xTB structures; (iii) sensitivity analysis of ranking parameters and re-ranking with the MD term removed; and (iv) additional short xTB-MD completion runs from partial stable trajectory seeds for selected systems.

The workflow was developed and executed through a conda-based Python environment. Then the calculations were executed on the CEDIA high-performance computing environment using the available Python Base/Singularity workflow and the nano_sim environment containing xTB 6.7.1. The computational environment used Python 3.14.6, NumPy, pandas, SciPy, Matplotlib, PyYAML, RDKit, Biopython, and tqdm. Semiempirical quantum chemical calculations and xTB-MD trajectories were performed with xTB 6.7.1. Density functional refinement was performed with ORCA 6.1.1.

The workflow was designed for candidate prioritization and uncertainty-aware hypothesis generation rather than for experimentally calibrated adsorption free energies, complete nanoparticle behavior, explicit-solvent molecular dynamics, or direct microbial-toxicity prediction. Therefore, the interpretation treats all xTB interaction values as relative descriptors within a reduced finite-patch model. Very large xTB magnitudes, nonconvergent ORCA target complexes, low-frame MD outputs, and sensitivity-dependent ranking changes were retained as explicit uncertainty flags rather than being discarded or interpreted as definitive material properties.

### 2.2. Construction of the Screening Library

The screening space was built from three finite oxide surface-patch models (TiO_2__patch, ZnO_patch, and CeO_2__patch), three surface ligands or surface states (glycerol, catechol, and citric_acid, plus the bare state), three hydrocarbon probes (hexane, toluene, and naphthalene), and two microbial-interface proxies (nag_proxy and surface_peptide_demo). The oxide seeds were provided as local .xyz structures and should be interpreted as reduced local models of oxide nanoparticle surfaces rather than as complete nanoparticle models. The ligands glycerol, catechol, and citric acid, the hydrocarbons hexane, toluene, and naphthalene, and the carbohydrate-like microbial proxy nag_proxy were generated from SMILES strings. The peptide-like microbial proxy surface_peptide_demo was generated from the FASTA sequence GGSTNQ.

This reduced representation was selected to enable controlled comparison of local oxide–ligand interfacial chemistry across all candidates under the same computational protocol. Accordingly, all descriptors reported in this work should be interpreted as relative screening descriptors within the studied candidate library. They are not intended to represent complete colloidal nanoparticle behavior, aggregation state, surface heterogeneity, dissolution, protein-corona formation, or full microbial-envelope response.

For all SMILES- and FASTA-derived species, RDKit generated 3D conformers with ETKDGv3, followed by hydrogen addition and force-field relaxation using UFF with a maximum of 1000 optimization iterations. Each prepared entity was written as an .xyz file and logged in an entity manifest containing the species identity, source representation, charge, multiplicity, and atom count. All entities used in the present study were neutral singlets.

The three oxide families were then combined with four surface states—bare, glycerol, catechol, and citric acid—to generate a 12-candidate library. For functionalized candidates, the workflow first identified the outward-facing surface site as the farthest metal atom from the nanoparticle center of mass, restricted to the corresponding surface symbol set (Ti, Zn, or Ce). The ligand anchor atom was selected as the first heteroatom in the order O, N, P, and S. The ligand was rotated to align its anchor vector with the outward surface direction and was then translated to an initial candidate–ligand separation of 2.1 Å. The bare state retained the oxide seed without surface addition. Candidate charge and multiplicity were assigned as

(1) $$q_{\mathrm{cand}}=q_{\mathrm{NP}}+q_{\mathrm{lig}}$$

(2) $$M_{\mathrm{cand}}=\max\left(M_{\mathrm{NP}},M_{\mathrm{lig}}\right)$$

where q is the total charge, and M is the spin multiplicity.

### 2.3. Generation of Candidate–Hydrocarbon and Candidate–Target Complexes

Each candidate was combined with the three hydrocarbon probes and the two microbial-interface proxies. Starting complexes were generated through a deterministic packing procedure that placed the analyte along three orthogonal directions, defined by the Cartesian unit vectors $+\hat{x}$, $+\hat{y}$, and $+\hat{z}$. The guest center of mass was placed at

(3) $$r_{\mathrm{guest}}^{\;0}=r_{\mathrm{host},\;\mathrm{COM}}+\hat{d}\;\left(R_{\mathrm{host}}+R_{\mathrm{guest}}+g\right)$$

where $r_{\mathrm{host},\;\mathrm{COM}}$ is the host center of mass, $\hat{d}\;$ is the packing direction, $R_{\mathrm{host}}$ and $R_{\mathrm{guest}}$ are the principal extents of the host and guest, and g = 3.0 g = 3.0 g = 3.0 Å is the prescribed gap. This strategy generated three poses per candidate–analyte pair, which yielded 180 starting complexes in total: 12 candidates × 5 analytes × 3 poses = 180 complexes.

Complex charges and multiplicities were assigned as

(4) $$q_{\mathrm{complex}}=q_{\mathrm{cand}}+q_{\mathrm{analyte}}$$

(5) $$M_{\mathrm{complex}}=\max\left(M_{\mathrm{cand}},M_{\mathrm{analyte}}\right)$$

The resulting complex manifest stored the candidate ID, analyte ID, role (hydrocarbon or target), pose number, charge, multiplicity, and .xyz path for downstream screening.

### 2.4. xTB Geometry Optimization and Binding-Energy Screening

The first screening layer used GFN2-xTB as implemented in xTB 6.7.1. Geometry optimizations were carried out with the keyword combination corresponding to --gfn 2 --opt tight --alpb water, and the calculations used six parallel threads. The solvent environment was represented by ALPB water. The workflow optimized all isolated entities, all functionalized candidates, and all prepared candidate–analyte complexes.

For each optimized complex, the binding energy was computed as

(6) $$\Delta E_{\mathrm{bind}}=E_{\mathrm{complex}}-E_{\mathrm{candidate}}-E_{\mathrm{analyte}}$$

where all terms are total electronic energies from the same xTB level of theory. Energies were converted from Hartree to kcal mol^−1^ using the factor 627.509474. Negative $\Delta E_{\mathrm{bind}}$ values correspond to a favorable association.

Because the workflow uses reduced oxide surface-patch models and semiempirical screening calculations, the resulting interaction energies were used as relative interaction-energy descriptors rather than as quantitative adsorption free energies. No entropic, zero-point, finite-temperature free-energy, explicit-solvent sampling, pH-dependent charge regulation, ionic-strength correction, dissolved organic matter competition, aggregation, or protein-corona correction was applied at this stage. Therefore, very large interaction magnitudes were interpreted as high-affinity or high-target-engagement warning signals within the reduced-cluster representation, especially when they occurred together with strong microbial-proxy penalties.

To address the possibility that extreme xTB values were influenced by mixed fallback energies, nonuniform convergence behavior, or reduced-cluster artifacts, the workflow added a clean GFN2-xTB/ALPB rescoring layer. This layer recomputed candidate, analyte, and complex energies using a consistent single-point protocol for 203 structures and 180 candidate–analyte complexes. The first clean rescore produced 146 successful structures and 111 complete complex binding-energy descriptors. Tier-1 rescue increased recovery to 169 successful structures and 146 complete complexes. Tier-2 rescue recovered all remaining structures, yielding 203/203 successful structures and 180/180 complete complex descriptors. However, the Tier-2 layer was not used to replace the original screening interpretation as a definitive adsorption ranking. Instead, it was retained as a robustness and uncertainty-tracking layer because several large-magnitude descriptors persisted after recovery.

The candidate interpretation, therefore, combines the original chemically interpretable screening trends, clean-rescore recovery status, descriptor-severity flags, ORCA refinement where available, MD frame-quality information, and sensitivity behavior. This procedure prevents extreme semiempirical descriptors from being interpreted as physical adsorption free energies while still preserving their diagnostic value as warning signals for strong or out-of-domain interfacial association.

### 2.5. Low-Temperature xTB-MD Perturbation Analysis and Frame-Quality Control

The MD layer was used as a short-timescale perturbation probe of candidate–target interfaces, not as an equilibrium molecular dynamics simulation. The original MD screen focused on the best xTB candidate–target complex for each candidate–target pair, yielding 24 candidate–target systems. These trajectories were propagated with GFN2-xTB MD at 277.15 K using NVT propagation, hmass = 4, shake = 2, and sccacc = 2.0. The original MD descriptors were target-fragment RMSD and contact persistence, computed with a 3.5 Å minimum-distance threshold.

In response to reviewer concerns about trajectories represented by only a few retained frames, the analysis added an explicit MD frame-quality classification. Candidate–target MD systems with at least 20 retained frames were classified as usable short-screening trajectories, whereas systems with 1–3 retained frames were classified as low-confidence MD outputs. The integrated ranking was recomputed with the MD-derived RMSD term removed to test whether low-frame MD systems controlled the final interpretation. This re-ranking was used as a robustness check rather than as a replacement for the original multi-criterion score.

Additional attempts were then made to extend selected xTB-MD systems. Direct extended-MD attempts were unstable for several reduced oxide–proxy complexes or produced trajectories with hard numerical flags, including SCC nonconvergence, lattice-point errors, emergency exits, or external interruption. These outcomes were retained as diagnostic evidence that the reduced finite-patch models should not be overinterpreted as stable explicit-solvent dynamical systems. Partial trajectories were harvested and classified, and only trajectories without hard instability flags or with external-interruption-only flags were considered for seed extraction.

Finally, three short xTB-MD completion trajectories were obtained from early frames of previously generated partial trajectories. These completion runs terminated normally and provided 50 analyzed frames per system. TiO_2_–glycerol with the peptide proxy was completed with GFN2-xTB gas-phase MD for 1.0 ps at 277.15 K and showed moderate target-proxy perturbation. TiO_2_–glycerol with the NAG proxy was also completed for 1.0 ps at 277.15 K but showed very high proxy displacement. ZnO–catechol with the peptide proxy was completed for 0.5 ps at 235 K and also showed very high proxy displacement. These completion trajectories were interpreted only as local short-timescale perturbation descriptors. They were not used as equilibrium MD, explicit-solvent sampling, toxicity evidence, or proof of microbial compatibility.

### 2.6. ORCA Refinement of the Shortlisted Candidates

The shortlist for DFT refinement was selected from the xTB–MD layer using a preliminary score that combined adsorption, target interaction, and structural perturbation. For each candidate, the preliminary score was defined as

(7) $$S_{\mathrm{prelim}}=\tilde{\left(-\Delta E_{\mathrm{hydro}}^{\mathrm{xTB}}\right)}-0.6\;\tilde{\left|\Delta E_{\mathrm{target}}^{\mathrm{xTB}}\right|}-0.4\;\tilde{\mathrm{RMSD}}$$

where x denotes min–max normalization over the candidate set. The workflow retained the top four candidates for ORCA refinement.

The four-candidate ORCA subset was selected as a refinement layer rather than as a complete DFT-level reranking of the full library. This choice was made because the 12-candidate library generated 180 candidate–analyte complexes, and full DFT treatment of every pose and target would have shifted the study from screening to exhaustive electronic-structure characterization. The selected subset, therefore, represents the most informative candidates according to the integrated xTB–MD score, while sensitivity analysis and clean-rescore uncertainty tracking were used to evaluate whether additional candidates could alter the interpretation.

ORCA input files were generated automatically from the optimized .xyz geometries using ORCA 6.1.1 with the keyword string: $r2SCAN-3c\;CPCM\left(Water\right)\;TightSCFOpt$.

The generated inputs allocated 2000 MB of memory per core and stored the full Cartesian coordinates for each job. For each shortlisted candidate, the workflow generated five ORCA job types:

- isolated candidate,
- isolated hydrocarbon partner,
- candidate–hydrocarbon complex,
- isolated microbial target, and
- candidate–target complex.

The hydrocarbon complex submitted to ORCA corresponded to the best xTB hydrocarbon-binding complex for that candidate. The target submitted to ORCA was selected from the MD stage as the target with the largest RMSD; when an MD endpoint was not available, the workflow fell back to the strongest target interaction identified in the xTB screen.

The ORCA binding energies were evaluated with the same expression used at the xTB stage, replacing xTB total energies with ORCA total energies.

ORCA refinement was used as a higher-level energetic check for the shortlisted systems, but the refinement layer was not treated as a complete reoptimization of the full candidate space. When ORCA calculations did not converge for a target complex, the corresponding target-binding result was treated as unresolved and was not used as definitive evidence of microbial-proxy perturbation profile. For such cases, interpretation relied on the combined xTB and MD screening profile while explicitly retaining the uncertainty associated with the unresolved refinement.

Unresolved ORCA calculations were treated as uncertainty flags and not as negative or positive evidence of microbial-proxy mildness. Two target-complex refinements remained unresolved: ZnO–citric acid with the NAG proxy and TiO_2_–glycerol with the peptide proxy. The ZnO–citric acid target complex showed SCF nonconvergence together with electronic-structure instability indicators, and the TiO_2_–glycerol peptide-proxy target complex also failed to converge after extended SCF cycling. These cases were therefore excluded from definitive ORCA target-binding interpretation and retained in the integrated evidence matrix as unresolved refinement outcomes. Resolved ORCA hydrocarbon or target energies were used only as a refinement layer for shortlisted systems and not as a complete re-ranking of the 12-candidate library.

### 2.7. Integrated Ranking and Pareto Analysis

The final ranking combined the xTB screen, MD descriptors, and ORCA refinement. When available, ORCA binding energies replaced the corresponding xTB binding energies for the refined candidates. The hydrocarbon contribution was converted into an adsorption axis:

(8) $$A=-\Delta E_{\mathrm{hydro}}$$

so that larger A values correspond to stronger hydrocarbon attraction.

Target aggression was converted into a target-binding excess penalty relative to a mild-interaction threshold of 25.0 kcal mol^−1^,

(9) $$P_{\mathrm{target}}=\frac{\max\left(0,\left|\Delta E_{\mathrm{target}}\right|-\Delta E_{\mathrm{mild}}\right)}{\Delta E_{\mathrm{mild}}};\Delta E_{\mathrm{mild}}=25.0\;\mathrm{kcal}\;\mathrm{mo}l^{-1}$$

The structural contribution was defined from the target RMSD and a soft structural limit of 2.0 Å,

(10) $$P_{\mathrm{RMSD}}=\frac{\mathrm{RMSD}}{{\mathrm{RMSD}}_{\mathrm{soft}}};\;{\mathrm{RMSD}}_{\mathrm{soft}}=2.0\;\backslash \mathrm{AA}$$

The total raw penalty was then written as

(11) $$P_{\mathrm{raw}}=P_{\mathrm{target}}+P_{\mathrm{RMSD}}$$

The final composite score used min–max normalized adsorption and penalty terms:

(12) $$S_{\mathrm{comp}}=\tilde{A}-w_{p}\;\tilde{P_{\mathrm{raw}}};w_{p}=0.65$$

where w_p_ is the penalty weight defined in the workflow configuration. This score rewards hydrocarbon association and favors mild target interaction and small structural perturbation.

The composite score was used as a prioritization metric and not as an absolute measure of material performance. Its purpose was to identify candidates occupying a favorable region of the adsorption–microbial-proxy tradeoff space. For this reason, the final interpretation emphasized candidate classes and experimentally testable leads rather than exact numerical score differences.

In parallel, the workflow identified Pareto-optimal candidates in the two-dimensional space (A, −Praw), which preserves the balance between adsorption and microbial-proxy perturbation profile without collapsing the problem into a single scalar. Final tables and figures were generated from this integrated ranking layer with Matplotlib.

### 2.8. Sensitivity Analysis and Final Evidence Classification

To evaluate the dependence of the final ranking on empirical scoring assumptions, the workflow added a sensitivity analysis over the target-binding threshold, RMSD soft limit, penalty weight, and inclusion or exclusion of the MD-derived RMSD term. The target-binding threshold was varied over 10, 15, 25, 35, 50, and 75 kcal mol−1; the RMSD soft limit was varied over 1.0, 2.0, 3.0, and 5.0 Å; the penalty weight was varied over 0.40, 0.50, 0.65, and 0.80; and rankings were recomputed both with and without the MD term. For each parameterization, candidates were re-ranked and summarized by median rank, mean rank, best rank, worst rank, top-3 frequency, and top-4 frequency.

The evidence matrix integrated original xTB descriptors, clean-rescore descriptors, xTB recovery status, ORCA availability, ORCA nonconvergence flags, MD frame quality, completed short-MD perturbation descriptors, and sensitivity behavior. Candidates were classified into interpretation classes rather than treated as a single definitive ranking. Primary experimentally testable candidates were defined as candidates with chemically interpretable original screening behavior and favorable microbial-proxy interaction profiles, even when clean-rescore uncertainty flags required cautious interpretation. Sensitivity-competitive secondary candidates were defined as candidates that appeared in top-ranked regions across multiple scoring parameterizations but lacked the same primary evidence profile. Warning cases were defined by extreme descriptor magnitudes, strong target engagement, low-confidence MD, unresolved refinement, or very high completed-MD perturbation. This classification strategy directly supports experimental prioritization while avoiding overclaiming whole-nanoparticle performance.

## 3. Results

### 3.1. Construction of the Oxide–Ligand Screening Space

**Figure 1:**
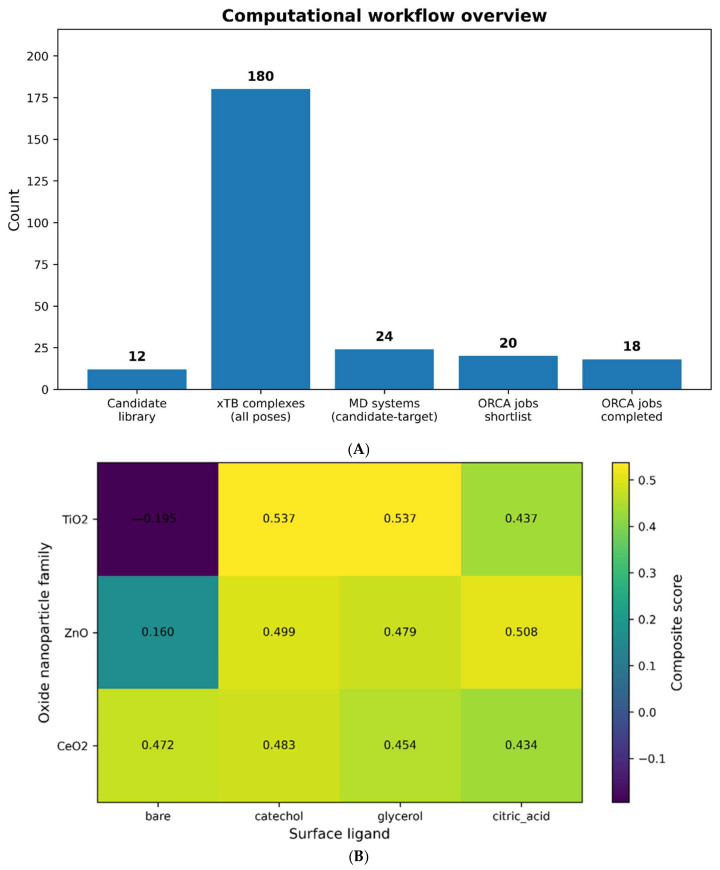
Workflow and design space of the oxide–ligand screening study. (**A**) Computational workflow used to evaluate oxide surface-patch candidates from initial structure preparation and xTB screening to short-timescale low-temperature MD, ORCA refinement, and integrated ranking. (**B**) Composite score matrix across oxide surface-patch families and surface ligands, showing the distribution of final screening performance across the 12-candidate library.

The study evaluates a rational oxide–ligand design space built around three finite oxide surface-patch model families, namely CeO_2_, TiO_2_, and ZnO, each represented by four surface states: bare, catechol, glycerol, and citric acid. This design generates a 12-candidate library that captures both oxide identity and surface chemistry as primary determinants of local interfacial behavior. The screening workflow then projects this candidate set against three hydrocarbon probes, hexane, toluene, and naphthalene, and two microbial-interface proxies, nag_proxy and surface_peptide_demo, thereby defining a dual-objective space centered on hydrocarbon association and microbial-proxy perturbation profile. Figure 1 summarizes the overall workflow and the distribution of candidate scores across oxide–ligand combinations. The composition of the candidate library and the main computational descriptors used to define the screening space are summarized in Table 1.

This design establishes the central objective of the study as the identification of oxide–ligand surface-patch interfaces that favor hydrocarbon association while preserving a mild interaction profile toward microbial-envelope-related proxy targets. The candidate matrix shows that the balance problem depends on the combination of oxide family and surface ligand rather than on oxide composition alone, which supports a comparative interpretation of the full library while avoiding whole-nanoparticle claims.

### 3.2. xTB Hydrocarbon Screening Across Oxide–Ligand Candidates

The xTB screening reveals a broad range of hydrocarbon-binding behaviors across the oxide–ligand library. Several candidates display very large binding magnitudes, particularly within the CeO_2_ and ZnO families, whereas the TiO_2_-derived candidates occupy a more moderate and chemically interpretable range. This pattern positions the hydrocarbon screen as an efficient first discriminator of adsorption-active interfaces and, at the same time, as a filter that highlights candidates requiring cautious interpretation when the calculated interaction strength becomes excessively large.

Within the TiO_2_ family, TiO_2__patch__catechol and TiO_2__patch__glycerol emerge as especially relevant. TiO_2__patch__catechol reaches its best xTB hydrocarbon association with naphthalene at pose 1, with a relative interaction-energy descriptor of −158.89 kcal/mol, whereas TiO_2__patch__glycerol reaches its best xTB hydrocarbon association with toluene at pose one, with a relative interaction-energy descriptor of −151.64 kcal/mol. These values define a moderate adsorption-associated regime compared with the much larger magnitudes calculated for several CeO_2_ and ZnO candidates, including CeO_2__patch__catechol (−3942.98 kcal/mol), CeO_2__patch__glycerol (−4108.94 kcal/mol), and zno_patch__citric_acid (−5339.88 kcal/mol). In line with the methodological scope of this work, these very large magnitudes are interpreted as high-affinity warning signals within the reduced surface-patch representation rather than as quantitative adsorption free energies. In this context, the TiO_2_-derived surfaces provide the most balanced adsorption-associated window for downstream microbial-proxy analysis. The best hydrocarbon-associated configuration identified for each candidate in the xTB screen is listed in Table 2.

**Figure 2:**
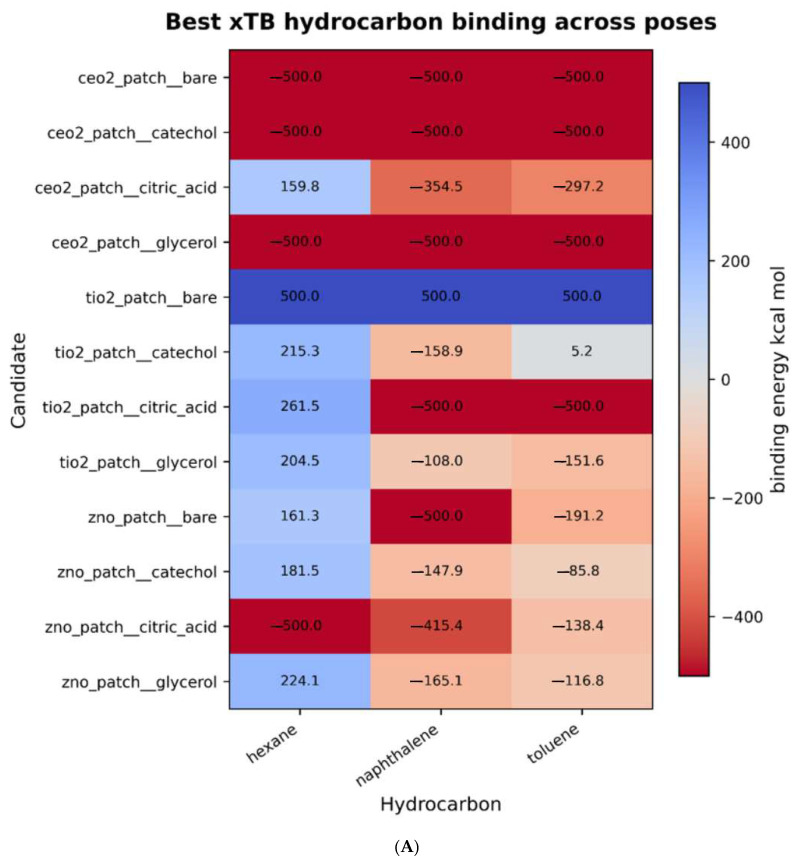

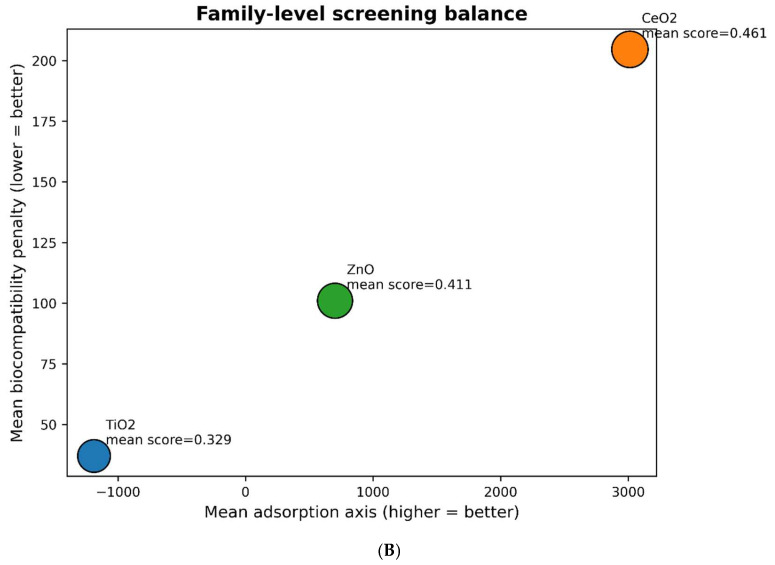
Hydrocarbon screening landscape across the oxide–ligand library. (**A**) Heatmap of the best xTB hydrocarbon-associated relative interaction-energy descriptors obtained for each candidate across the tested hydrocarbons and poses. The color scale is used for comparative visualization and should not be interpreted as a quantitative free-energy scale for extreme values. (**B**) Family-level tradeoff map summarizing the balance between adsorption-related performance and penalty-related terms across the main oxide surface-patch families.

The heatmap and the family-level tradeoff plot presented in Figure 2 clarify this distribution at two scales. The hydrocarbon heatmap resolves the preferred analyte–candidate combinations, whereas the family-level plot shows that TiO_2_ occupies the most favorable compromise region when adsorption strength is considered together with the downstream penalty-based ranking. This combined view supports the decision to retain TiO_2_-centered candidates as the most mechanistically informative branch of the screening space.

### 3.3. Microbial-Proxy Interaction Screening and the Emergence of a Microbial-Proxy Perturbation Filter

The microbial-proxy screen transforms the interpretation of the adsorption results by revealing how strongly each candidate engages with envelope-relevant targets. This second screening axis identifies candidates that preserve a gentle interfacial profile and separates them from candidates that associate strongly with microbial proxies. In practical terms, the microbial filter elevates selectivity over raw adsorption magnitude and defines the central ranking logic of the study.

The strongest evidence for this filtering step comes from the contrast between TiO_2_-derived candidates and several CeO_2_ and ZnO candidates. TiO_2__patch__catechol shows a worst-target xTB relative interaction-energy descriptor of −9.77 kcal/mol against surface_peptide_demo, and TiO_2__patch__glycerol shows a worst-target xTB relative interaction-energy descriptor of −16.71 kcal/mol against nag_proxy. These values represent the mildest target-interaction regime in the full library. In contrast, CeO_2__patch__catechol reaches −6124.31 kcal/mol against nag_proxy, CeO_2__patch__glycerol reaches −6706.25 kcal/mol against nag_proxy, and zno_patch__bare reaches −7782.18 kcal/mol against surface_peptide_demo. These extreme target-associated magnitudes were interpreted as high target-engagement warning signals rather than as quantitatively reliable binding energies. The microbial-proxy screen therefore prioritizes TiO_2__patch__catechol and TiO_2__patch__glycerol within the balance problem, while repositioning several strongly adsorbing candidates as high-penalty contrast interfaces. The strongest microbial-proxy interaction identified for each candidate, together with its qualitative interpretation, is reported in Table 3.

**Figure 3:**
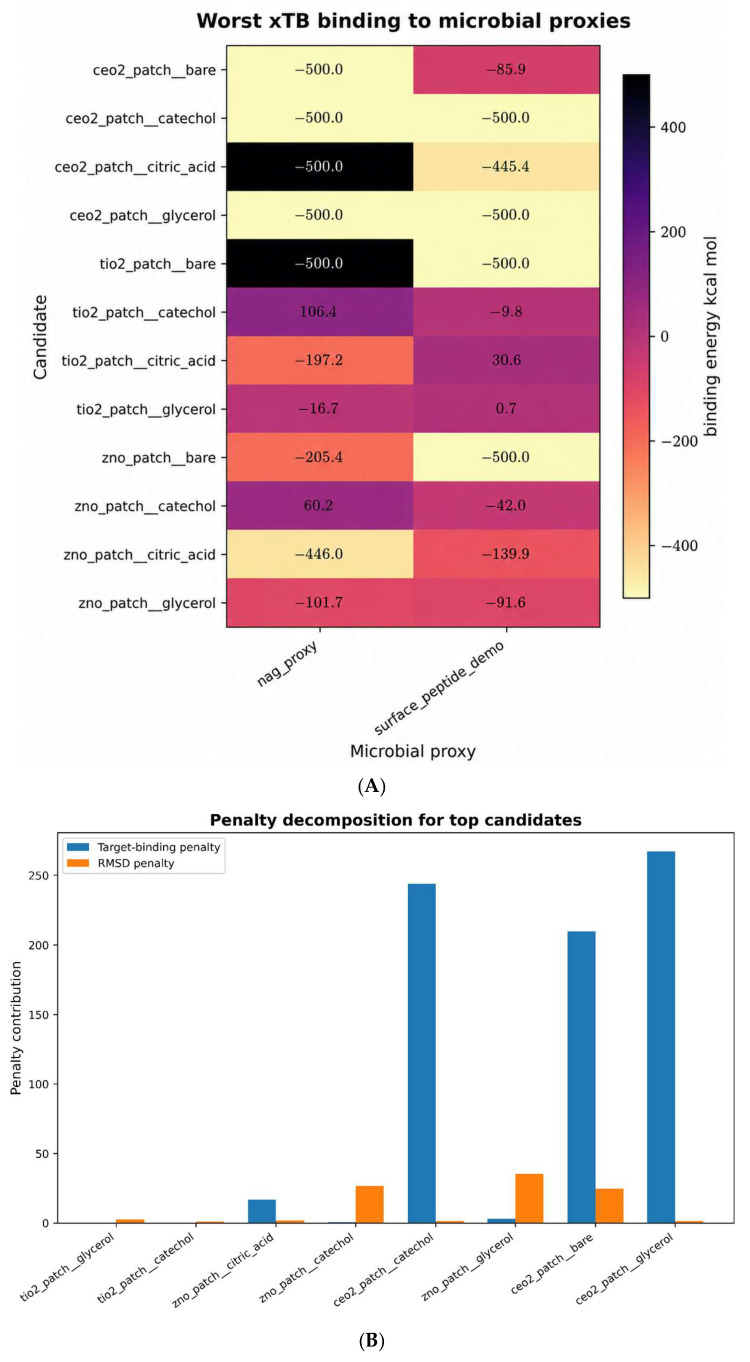

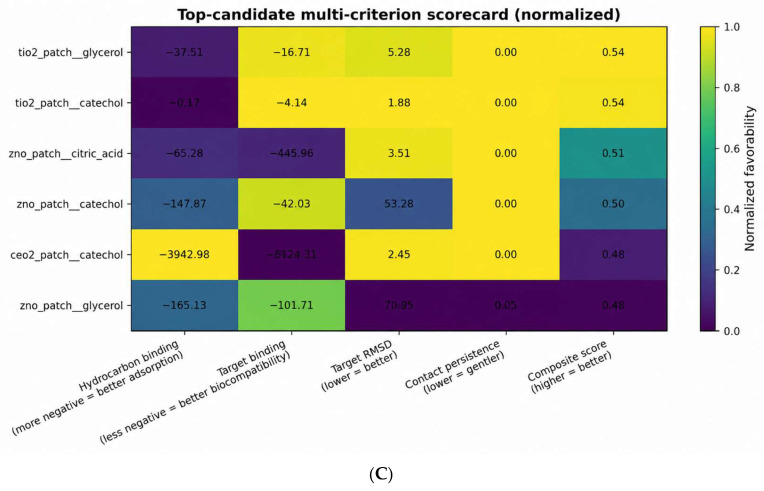
Microbial-proxy interaction patterns and penalty structure of the leading candidates. (**A**) Heatmap of the strongest xTB microbial-proxy interaction identified for each candidate. (**B**) Decomposition of the main penalty contributions among the top-ranked candidates. (**C**) Normalized multi-criterion scorecard summarizing the performance of the leading candidates across adsorption, microbial-proxy interaction, structural perturbation, and final composite score.

Figure 3 visualizes this shift. The target-binding heatmap displays the full penalty landscape, the penalty breakdown resolves how target binding and RMSD contribute to the composite penalty, and the top-candidate scorecard highlights how the leading candidates maintain favorable values across multiple descriptors simultaneously. Together, these plots define the microbial-proxy filter as the decisive interpretive step of the study.

### 3.4. MD Frame-Quality Audit and Completed Short Perturbation Trajectories

The MD layer was revised from a simple short-trajectory descriptor into a frame-quality and perturbation-audit layer. The original MD screen generated 24 candidate–target systems, but the retained trajectory quality was heterogeneous. Systems with at least 20 retained frames were treated as usable short-screening trajectories, whereas systems represented by only 1–3 frames were classified as low-confidence MD outputs. This distinction was introduced because low-frame systems cannot support statistically meaningful contact-persistence or RMSD trends by themselves.

The frame-quality audit showed that several initially attractive TiO_2_ systems had low-frame MD outputs in the original screen, whereas some ZnO and CeO_2_ systems had more usable frame counts but stronger perturbation or target-engagement profiles. To test whether the low-frame MD term controlled the final prioritization, the ranking was recomputed with the RMSD-derived MD term removed. This robustness check showed that ZnO–catechol and ZnO–glycerol remained competitive under several parameterizations, whereas TiO_2_–catechol and TiO_2_–glycerol should be interpreted as experimentally testable TiO_2_-centered hypotheses rather than as uniquely dominant candidates under every scoring assumption.

Additional direct extended-MD attempts were then performed for selected systems. These attempts confirmed that several reduced oxide–proxy complexes were numerically or structurally unstable under longer xTB-MD conditions, producing SCC nonconvergence, lattice-point failures, emergency exits, or externally interrupted trajectories. A harvest analysis identified 66 trajectory attempts: 57 were not usable, eight were trajectory-but-flagged cases, and only one minimal trajectory contained two frames without hard flags. These results support a cautious interpretation of the MD layer as a perturbation diagnostic rather than as equilibrium dynamics.

To obtain completed short trajectories for selected systems, early frames from partial trajectories with no hard instability flags were used as seeds for short completion MD. Three completion trajectories terminated normally and provided 50 analyzed frames each. TiO_2_–glycerol with the peptide proxy completed a 1.0 ps GFN2-xTB gas-phase trajectory at 277.15 K and showed a mean target RMSD of 2.73 Å, a maximum RMSD of 2.80 Å, no persistent contact, and a mean minimum distance of 11.31 Å. This result indicates moderate microbial-proxy perturbation under the completed short trajectory. In contrast, TiO_2_–glycerol with the NAG proxy completed a 1.0 ps trajectory but showed very high proxy displacement, with a mean RMSD of 63.50 Å. ZnO–catechol with the peptide proxy completed a 0.5 ps trajectory at 235 K and also showed very high proxy displacement, with a mean RMSD of 53.63 Å. These completed trajectories therefore support TiO_2_–glycerol as a peptide-proxy-moderate case, while they caution against interpreting all sensitivity-competitive candidates as dynamically mild (Table 4).

The completed MD trajectories reinforce the interpretation of the workflow. MD does not validate microbial compatibility, but it helps separate moderate perturbation from strong proxy displacement among selected candidates. Accordingly, the final ranking uses MD as qualitative perturbation evidence and explicitly distinguishes completed short-MD results from equilibrium molecular dynamics or toxicity assays.

### 3.5. ORCA Refinement of the Shortlisted Candidates

ORCA refinement focuses the analysis on the four shortlisted candidates selected from the integrated xTB–MD screen: TiO_2__patch__catechol, TiO_2__patch__glycerol, TiO_2__patch__citric_acid, and zno_patch__citric_acid. Eighteen ORCA jobs terminate normally, and two target-complex jobs remain unconverged, namely the zno_patch__citric_acid complex with nag_proxy and the TiO_2__patch__glycerol complex with surface_peptide_demo. This success rate provides a strong refinement basis for the shortlist while preserving a transparent record of the two unresolved target complexes.

The resolved ORCA data are consistent with the TiO_2_-centered interpretation, while the unresolved target-complex calculations preserve uncertainty for part of the shortlist. TiO_2__patch__glycerol yields the strongest resolved ORCA hydrocarbon-association value among the shortlisted candidates, with −37.51 kcal/mol for the toluene complex. TiO_2__patch__catechol yields a much weaker hydrocarbon-association value, −0.17 kcal/mol for the naphthalene complex, and the mildest resolved ORCA target-interaction value, −4.14 kcal/mol for the surface_peptide_demo complex. The zno_patch__citric_acid yields −65.28 kcal/mol for the hexane complex, while its target complex remains unresolved. TiO_2__patch__citric_acid shows an unfavorable ORCA profile, with +34.34 kcal/mol for the hydrocarbon term and +1204.99 kcal/mol for the target term, and therefore exits the lead-candidate space.

**Figure 4:**
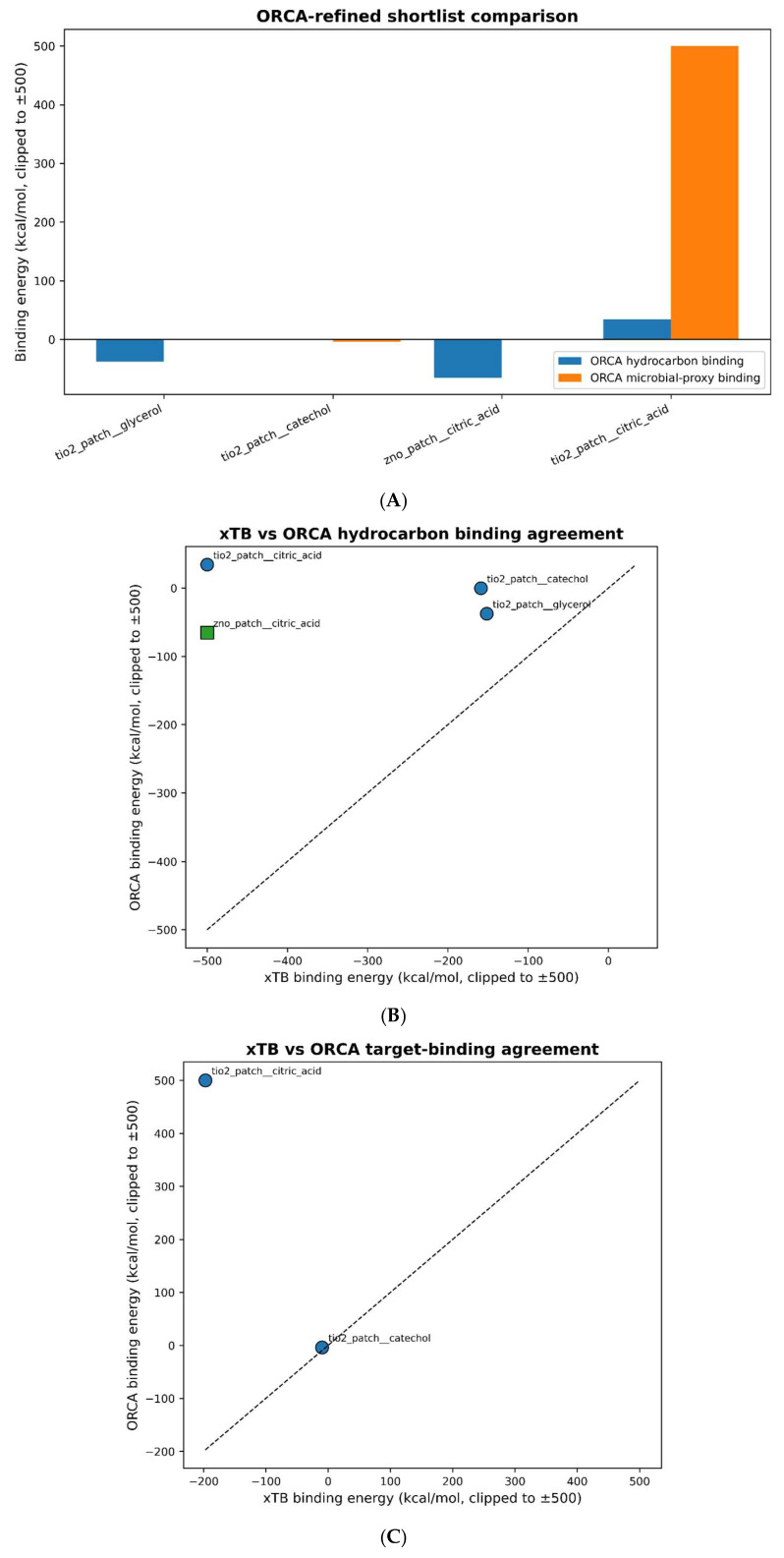
ORCA refinement of the shortlisted candidates. (**A**) Comparison of ORCA-refined hydrocarbon-binding and microbial-proxy binding values for the shortlisted candidates. (**B**) Relationship between xTB and ORCA hydrocarbon-binding values. (**C**) Relationship between xTB and ORCA microbial-proxy binding values for the refined subset.

The comparison plots in Figure 4 show that ORCA refinement provides a higher-level energetic check for the resolved portion of the shortlist. The ORCA shortlist comparison directly contrasts hydrocarbon and microbial-proxy energetics, while the xTB–ORCA agreement plots situate the refined values against the lower-cost screening layer. Because two target-complex refinements remained unresolved, the ORCA layer was interpreted as partial confirmatory evidence rather than as a complete re-ranking of the shortlist. Within that scope, the resolved ORCA results support a final interpretation centered on two complementary TiO_2_-derived interfaces: one adsorption-oriented and one interface-conservative.

### 3.6. Integrated Ranking and Identification of Lead Candidates

The integrated ranking resolves the full screening problem into a two-lead prioritization scenario. TiO_2__patch__glycerol occupies the top position with a composite score of 0.5373 and remains Pareto-optimal, while TiO_2__patch__catechol follows closely with a composite score of 0.5366 and also remains Pareto-optimal. Because the score was designed as a relative prioritization metric, these small numerical differences should not be overinterpreted as absolute performance gaps. Instead, the two candidates are best interpreted as complementary lead profiles within the adsorption–microbial-proxy perturbation profile tradeoff. The final integrated ranking and the decision labels assigned to each candidate are presented in Table 5.

TiO_2__patch__glycerol represents the adsorption-oriented lead candidate. It combines an ORCA hydrocarbon-binding value of −37.51 kcal/mol with an xTB target-interaction descriptor of −16.71 kcal/mol and a target RMSD of 5.28 Å. This profile supports a candidate that engages the hydrocarbon phase more strongly while maintaining a moderate microbial-proxy penalty within the screening model. TiO_2__patch__catechol represents the interface-conservative lead candidate. It combines a hydrocarbon-binding value of −0.17 kcal/mol with a resolved target-binding value of −4.14 kcal/mol and a target RMSD of 1.88 Å. This profile supports a conservative interface whose strength lies in mild resolved target interaction and low early structural perturbation, while still requiring experimental validation.

The remaining candidates define secondary or contrast classes. The zno_patch__citric_acid retains a relatively high composite score of 0.5079 and remains Pareto-optimal, yet its xTB magnitudes are unusually large and one ORCA target-complex job remains unresolved, which positions it as a contrast case rather than a primary lead. The zno_patch__catechol also remains Pareto-optimal, although its RMSD value of 53.28 Å places it outside the most attractive interface regime. The CeO_2_ candidates populate a high-adsorption, high-penalty region and therefore contribute more strongly as mechanistic contrasts than as lead interfaces.

**Figure 5:**
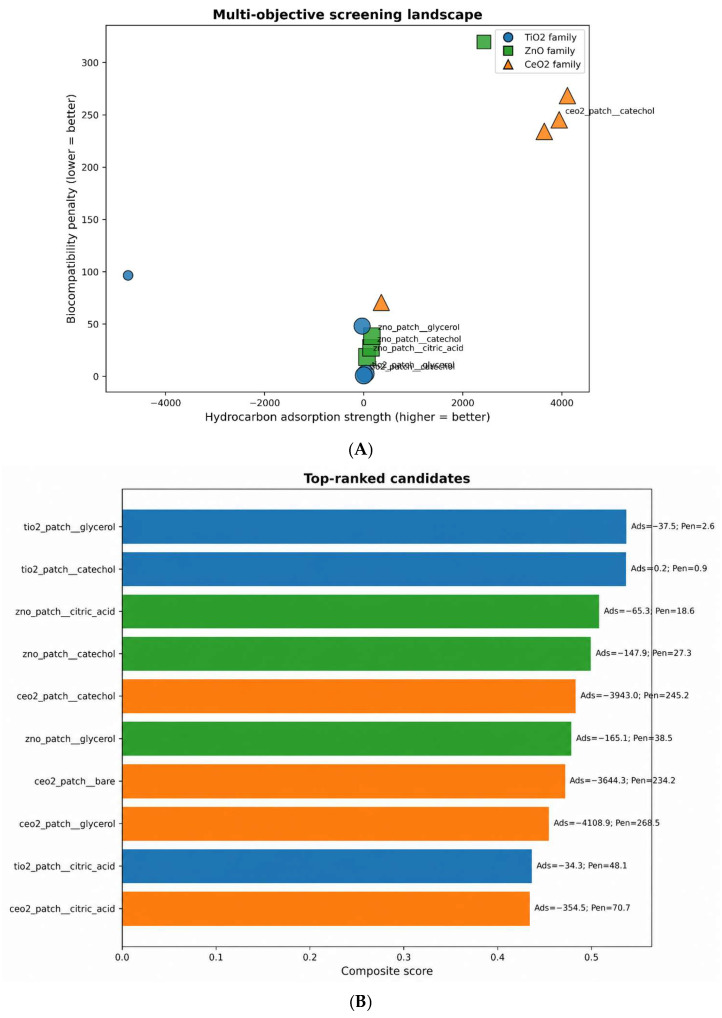
Integrated ranking of adsorption–biocompatibility performance. (**A**) Pareto landscape summarizing the balance between adsorption-oriented and penalty-oriented dimensions across the screened candidates. (**B**) Ranking of the top candidates according to the final composite score.

Figure 5 summarizes this integrated interpretation. The Pareto landscape and the ranked composite-score plot define the full balance space, while the main multi-panel figure condenses the ranking narrative into a single visual statement suitable for the main text. Together, these plots support the final selection of TiO_2__patch__glycerol and TiO_2__patch__catechol as the two lead candidates of the study.

### 3.7. Structural Exemplars of the Selected Interfaces

The structural figures serve as visual exemplars of the two lead profiles and one contrast case. The TiO_2_ storyboard captures the progression from isolated candidate to hydrocarbon-associated complex and, subsequently, to the microbial-proxy endpoint. This visual sequence illustrates the central screening interpretation of the study: TiO_2_-derived interfaces occupy the most favorable relative balance between hydrocarbon association and microbial-proxy perturbation profile within the evaluated oxide–ligand surface-patch space.

The TiO_2__patch__catechol panels represent the conservative interface solution. The isolated structure shows a compact TiO_2_-derived surface decorated by a catechol shell. The hydrocarbon complex preserves a compact adsorption geometry, and the microbial-interface endpoint remains visually consistent with the low target RMSD and low target-binding penalty identified in the quantitative analysis. The TiO_2__patch__glycerol panels represent the adsorption-balanced solution. The hydrocarbon complex highlights a stronger adsorption profile, and the endpoint structure remains compatible with a moderate perturbation regime. The ZnO–citric acid panels retain value as a contrast case because they visualize a candidate that combines strong hydrocarbon engagement with a more ambiguous microbial-interface profile.

## 4. Discussion

### 4.1. Interfacial Selectivity as the Central Screening Principle

The results reposition this study as an uncertainty-aware interfacial screening framework rather than as a direct validation of nanoparticle-assisted bioremediation. The most important finding is that raw hydrocarbon affinity is not sufficient for candidate selection. Several oxide–ligand systems displayed very large xTB hydrocarbon-association descriptors, but the same systems also showed strong microbial-proxy interaction penalties, extreme clean-rescore magnitudes, unresolved refinement behavior, low-confidence MD outputs, or very high proxy displacement in completed short xTB-MD trajectories. This pattern supports interfacial selectivity as the central design principle: a useful candidate should promote hydrocarbon association while avoiding excessive interaction with microbial-envelope-related proxies.

The proposed candidates should also be interpreted relative to existing oxide-based bioremediation observations. Prior experimental work has shown that Fe_2_O_3_ and ZnO nanoparticles can accelerate crude-oil biodegradation when combined with hydrocarbon-degrading bacteria [7], indicating that oxide nanomaterials can influence hydrocarbon availability and microbial degradation under suitable conditions. However, the same broader literature also shows that ZnO and CeO_2_ systems may interact strongly with bacterial envelopes, modify membrane permeability, or express antibacterial behavior depending on exposure conditions and surface chemistry [28,29,30]. The present study, therefore, does not claim that TiO_2_–catechol or TiO_2_–glycerol are superior validated materials. Instead, it identifies them as chemically plausible experimental hypotheses because they occupy a more selective region of the reduced screening space: moderate hydrocarbon association, comparatively mild microbial-proxy descriptors in the original screening layer, and explicit uncertainty flags for cases requiring validation. The practical advantage of these candidates is not proven performance, but a more defensible starting point for low-temperature experiments than candidates selected only by maximum adsorption strength.

This interpretation is consistent with the biological structure of low-temperature hydrocarbon biodegradation. Cold-adapted and psychrotolerant microorganisms can degrade hydrocarbons under low-temperature conditions, including polar and cold marine systems [1,3,4,5,12,15]. However, biodegradation is not controlled by catabolic potential alone. Hydrocarbon bioavailability, substrate encounter, and interfacial transport strongly influence the actual degradation rate [6]. Therefore, a computational material-screening workflow for cold bioremediation should not prioritize adsorption strength alone; it should evaluate whether the same interface that captures or associates with hydrocarbons may also perturb microbial-envelope-related structures.

The present results extend earlier nano-assisted bioremediation observations by separating adsorption activity from microbial proxy perturbation. Prior work showed that oxide nanoparticles, including ZnO- and Fe_2_O_3_-based systems, can enhance crude-oil biodegradation in bacterial systems [7]. At the same time, nanoparticle association with bacterial envelopes depends on material identity, surface chemistry, charge distribution, and local contact geometry [8,9,10,31,32,33,34,35,36,37,38]. The present workflow connects these two ideas into a single selection logic. The preferred oxide–ligand interface is not necessarily the one with the strongest hydrocarbon descriptor, but the one that occupies a more balanced region between hydrocarbon association, microbial-proxy mildness, structural perturbation, and computational uncertainty.

### 4.2. Functionalized TiO_2_ Interfaces as Primary Experimental Hypotheses

The TiO_2_-centered candidates remain the most coherent primary experimental hypotheses, but the interpretation is more cautious than the original two-lead statement. TiO_2_–catechol and TiO_2_–glycerol are retained as primary experimentally testable candidates because their original screening profiles combined chemically interpretable hydrocarbon association with comparatively mild microbial-proxy interaction descriptors. However, both candidates also carry uncertainty flags in the clean-rescore layer. Therefore, they should not be described as validated biocompatible materials. They should be described as experimentally testable interface hypotheses.

TiO_2_–catechol represents the interface-conservative hypothesis. Its original microbial-proxy interaction profile was mild, and the resolved ORCA target-associated result supported a comparatively low target-interaction descriptor. This behavior is chemically plausible because catechol forms stable and structurally ordered complexes with TiO_2_ surfaces, including chelated adsorption geometries and interfacial charge-transfer states [21,22,35,36,39]. In the context of the present finite-patch workflow, this suggests that catechol may moderate local surface reactivity while preserving a chemically meaningful oxide–organic junction. Nevertheless, the original MD output for TiO_2_–catechol had low frame confidence, and the clean-rescore layer produced extreme descriptor flags. For this reason, TiO_2_–catechol should be discussed as a conservative and experimentally testable TiO_2_ hypothesis rather than as a definitively stable interface.

TiO_2_–glycerol represents the adsorption-oriented TiO_2_ hypothesis. It retained hydrocarbon-association relevance in the original screening and showed moderate perturbation against the peptide proxy in the completed short xTB-MD trajectory. Specifically, the completed 1.0 ps GFN2-xTB gas-phase trajectory at 277.15 K provided 50 analyzed frames, with a mean target RMSD of 2.73 Å, a maximum RMSD of 2.80 Å, zero contact persistence, and a mean minimum distance of 11.31 Å. This result supports a moderate peptide-proxy perturbation profile under the completed short-trajectory conditions. However, TiO_2_–glycerol also showed very high displacement against the NAG proxy, with a mean RMSD of 63.50 Å in the corresponding completed trajectory. The ligand, therefore, appears proxy-dependent. It cannot be interpreted as generally mild toward all microbial-interface motifs.

Together, TiO_2_–catechol and TiO_2_–glycerol define two complementary experimental directions. TiO_2_–catechol is more suitable as an interface-conservative hypothesis, whereas TiO_2_–glycerol is more suitable as an adsorption-oriented hypothesis with proxy-dependent perturbation behavior. This distinction is important for experimental planning because the two candidates should not be evaluated with the same expected mechanism. TiO_2_–catechol should be tested for mildness of microbial-envelope contact, whereas TiO_2_–glycerol should be tested for whether improved hydrocarbon association can be achieved without excessive disruption of specific microbial-interface components.

### 4.3. Sensitivity-Competitive ZnO Candidates and the Limits of a Rigid Two-Lead Interpretation

The sensitivity analysis changes the interpretation of the ranking. The original manuscript emphasized TiO_2_–catechol and TiO_2_–glycerol as the two principal leads. The analysis keeps them as primary experimentally testable TiO_2_ hypotheses, but it no longer supports presenting them as the only relevant candidates under all scoring assumptions. ZnO–catechol and ZnO–glycerol emerged as sensitivity-competitive secondary candidates. ZnO–catechol showed a median sensitivity rank of 2.5 and a top-4 frequency of 0.8125, whereas ZnO–glycerol showed a median sensitivity rank of 3.5 and a top-4 frequency of 0.5208. This indicates that these ZnO systems occupy a competitive region of the multi-criterion space when ranking assumptions are varied.

This outcome is scientifically useful because it prevents the workflow from becoming a rigid score-maximization exercise. ZnO–catechol and ZnO–glycerol should not replace the TiO_2_-centered primary interpretation, but they provide secondary experimental hypotheses that should be considered in validation studies. Their relevance comes from ranking robustness rather than from a complete absence of uncertainty. ZnO–catechol, for example, completed a short 0.5 ps GFN2-xTB trajectory at 235 K with 50 analyzed frames, but the mean RMSD against the peptide proxy was 53.63 Å, indicating very high proxy displacement. This result suggests that ZnO–catechol is computationally competitive in static and sensitivity-based ranking, but it should be treated cautiously in dynamic proxy-perturbation terms.

The broader ZnO literature supports this caution. ZnO nanoparticles can damage bacteria under dark conditions through direct surface contact, Zn^2+^ release, membrane permeability changes, and metabolic disruption [28]. Therefore, ZnO-centered candidates should be interpreted as experimentally interesting but not automatically mild. The present results classify ZnO–catechol and ZnO–glycerol as sensitivity-competitive secondary candidates, while ZnO–citric acid is better treated as a warning or contrast case because of stronger target-associated descriptors and unresolved target-complex refinement. This distinction directly addresses the need to separate computational ranking from biological validation.

### 4.4. High-Magnitude CeO_2_ and ZnO Cases as Warning Interfaces

Several CeO_2_- and ZnO-based systems displayed very large xTB descriptor magnitudes. In the original version, these values risked being interpreted as very strong adsorption. The interpretation is more conservative. Very large xTB interaction magnitudes are treated as high-affinity or high-target-engagement warning signals within the reduced finite-patch representation, not as experimentally calibrated adsorption free energies. This distinction is essential because the models do not include entropy, explicit-solvent sampling, pH-dependent charge regulation, ionic strength, aggregation, dissolution, ligand-shell disorder, protein-corona formation, or complete nanoparticle morphology.

The CeO_2_ candidates define an upper boundary of the adsorption–proxy-perturbation problem. Several CeO_2_-based systems combined large hydrocarbon-associated magnitudes with strong microbial-proxy penalties. This pattern is consistent with the known biological complexity of cerium oxide nanoparticles. CeO_2_ nanoparticles can alter bacterial outer-membrane permeability and may act as antimicrobial adjuvants under specific conditions [29]. More broadly, cerium oxide nanomaterials may express benign, antioxidant, or antimicrobial behavior depending on redox state, surface chemistry, size, and biological context [30]. Therefore, the CeO_2_ outliers in this study should not be considered failed candidates. They are more appropriately interpreted as overactive or high-engagement interfaces that help define the upper limit beyond which adsorption may stop being useful for microbial-facing bioremediation design.

The same reasoning applies to high-magnitude ZnO systems. Strong association descriptors may be attractive from a hydrocarbon-capture perspective, but strong microbial-proxy engagement can become undesirable if it indicates excessive envelope interaction. In practical terms, the warning cases demonstrate why a two-axis or multi-objective screening framework is necessary. A single adsorption score would incorrectly favor some high-magnitude systems. The integrated evidence matrix, by contrast, preserves these systems as contrast cases that define the limits of acceptable interfacial selectivity.

### 4.5. Microbial-Envelope Proxies and Interpretation of the MD Layer

The microbial-proxy layer is justified by bacterial-envelope chemistry, but it must be interpreted within its reduced scope. Gram-negative bacterial envelopes contain lipopolysaccharide-rich outer-membrane regions, peptidoglycan-associated components, proteins, and chemically heterogeneous hydrated interfaces [24,25,26,27,28,29,30,31]. Nanoparticle association with Gram-negative outer membranes depends strongly on LPS density and structure [9], and oxide nanoparticles can interact with bacterial cell-envelope biomolecules, including signals associated with proteins, phospholipids, wall-related components, and LPS-rich surfaces [10]. These observations support the central premise of the workflow: microbial-facing target interaction must be screened directly and cannot be inferred from hydrocarbon association alone.

The NAG and GGSTNQ proxies are therefore useful as simplified molecular representatives of carbohydrate-rich and peptide-like envelope-related motifs. However, they are not complete bacterial-envelope models. They do not reproduce the outer membrane, LPS architecture, peptidoglycan network, membrane proteins, extracellular polymeric substances, cell curvature, active transport, metabolism, or community-level biodegradation. For that reason, the manuscript avoids using “biocompatibility” as a direct result. The safer interpretation is “microbial-proxy perturbation profile” or “microbial-interface proxy response.”

The MD layer must be interpreted with the same caution. The original 5 ps MD outputs were heterogeneous in frame quality, and several candidate–target systems retained only 1–3 frames. The frame-quality audit and MD-term-removal ranking show that these trajectories should not be used as converged dynamical evidence. The completed short xTB-MD trajectories improve the evidentiary basis by providing three normally terminating trajectories with 50 analyzed frames each. However, they remain short gas-phase xTB-MD perturbation probes generated from partial stable seeds. They are not explicit solvent equilibrium simulations, toxicity assays, or direct evidence of microbial compatibility.

This cautious MD interpretation is consistent with the broader molecular simulation literature on bacterial envelopes and solvent-dependent biomolecular behavior. The bacterial envelope must be treated as a multilayered and chemically heterogeneous barrier in molecular simulations [27]. In addition, solvent composition and evaporation-related changes can affect protein structural stability during molecular dynamics simulations, as shown for lysozyme in DMSO-containing electrospray-related simulations [40]. Although that system is not a bioremediation model, it reinforces the general point requested by the reviewer: local solvent environment, droplet behavior, and protein/peptide stability can alter biomolecular structure and should not be ignored in future simulations. Therefore, explicit-solvent and larger-scale envelope models are necessary before making stronger claims about microbial response.

### 4.6. Methodological Significance of the Uncertainty-Aware Workflow

The main methodological contribution of this study is the integration of screening, rescoring, sensitivity testing, and uncertainty classification in a single prioritization workflow. The initial xTB layer provided the throughput needed to evaluate 180 candidate–analyte complexes. The clean GFN2-xTB/ALPB rescore and Tier-1/Tier-2 rescue layers addressed reviewer concerns about convergence and extreme interaction magnitudes. The ORCA layer provided higher-level refinement for selected systems while explicitly retaining unresolved target-complex calculations as uncertainty flags. The MD layer was revised from a simple perturbation descriptor into a frame-quality and completion-trajectory audit. The sensitivity analysis then tested whether the ranking depended excessively on empirical scoring parameters.

This structure is stronger than the original ranking because it does not hide instability, nonconvergence, or out-of-domain descriptor behavior. Instead, it converts those outcomes into evidence categories. Primary candidates are not defined merely by high score, but by chemically interpretable original behavior, moderate or mild microbial-proxy descriptors, and experimentally useful hypotheses. Secondary candidates are defined by sensitivity and competitiveness. Warning cases are defined by extreme descriptors, unresolved target-complex refinement, low-confidence MD, or high completed-MD perturbation. This classification is better aligned with the actual uncertainty of the computational workflow.

The workflow also fits broader materials-screening logic for oxide systems. TiO_2_, ZnO, CeO_2_, Fe_2_O_3_, SiO_2_, Al_2_O_3_, and WO_3_ belong to a broad family of functional oxide materials whose performance is strongly governed by surface chemistry and interfacial environment [39]. Therefore, oxide identity alone cannot determine suitability for bioremediation. Ligand chemistry, local hydration, charge distribution, aggregation state, and biological contact mode are equally important. The present study contributes to this field by showing how a reduced molecular workflow can identify which oxide–ligand combinations deserve experimental testing first.

### 4.7. Limitations and Experimental Validation Requirements

Several limitations define the scope of the present results. First, the oxide systems were represented by finite surface-patch models rather than complete nanoparticles. They do not include particle size distributions, exposed crystalline facets, surface defects, hydroxylation heterogeneity, dissolution, aggregation, ligand-shell disorder, or colloidal stability. Therefore, the results describe local oxide–ligand interface behavior, not complete nanoparticle behavior.

Second, the xTB interaction-energy values are relative screening descriptors. They are not adsorption-free energies. They do not include entropic corrections, zero-point corrections, explicit-solvent free-energy sampling, finite-temperature ensemble averaging, pH-dependent charge regulation, ionic-strength effects, dissolved organic matter, competing ions, biosurfactants, extracellular polymeric substances, or hydrocarbon mixtures. Extreme values are therefore best interpreted as warning signals for strong or out-of-domain local interactions, not as physically exact adsorption strengths.

Third, the microbial-interface proxies are chemically meaningful but simplified. The NAG proxy and GGSTNQ peptide proxy cannot reproduce the full Gram-negative outer membrane, LPS architecture, peptidoglycan network, membrane proteins, cell-wall mechanics, or living-cell response. This limitation is especially important because nanoparticle–bacterial interactions depend on nanoscale contact geometry, local charge presentation, and membrane composition [9,10,30,31,38].

Fourth, the completed short MD trajectories are perturbation probes only. They help distinguish moderate proxy perturbation from very high proxy displacement in selected systems, but they do not establish equilibrium stability. They also do not represent explicit-solvent dynamics, live-cell toxicity, biodegradation enhancement, or field performance. Future work should use explicit-solvent simulations, hydrated and hydroxylated surface models, pH- and ionic-strength-dependent charge states, larger nanoparticle models, and complete bacterial-envelope representations.

Solvent effects are particularly important for the interpretation of the MD layer. The completed trajectories reported here were short gas-phase xTB-MD perturbation probes obtained from partial stable seeds; therefore, they cannot reproduce hydration-shell rearrangement, droplet evaporation, solvent clustering, or solvent-mediated peptide/protein stabilization. This limitation is relevant because recent MD work on lysozyme showed that solvent composition and evaporation behavior can modulate protein secondary structure, hydrogen-bond patterns, salt bridges, and conformational stability during droplet-related processes [40].

Fifth, ORCA refinement was limited to a shortlist, and two target-complex refinements remained unresolved. The unresolved ZnO–citric acid/NAG and TiO_2_–glycerol/peptide-proxy refinements should be treated as uncertainty flags, not as evidence of either mildness or incompatibility. This point is central to the interpretation because the study is intended to support prioritization, not definitive validation.

From a translational perspective, the next step is experimental validation. TiO_2_–catechol and TiO_2_–glycerol should be tested as primary TiO_2_-centered hypotheses, while ZnO–catechol and ZnO–glycerol should be retained as sensitivity-competitive secondary candidates. Validation should quantify hydrocarbon removal, bacterial growth, membrane integrity, surface charge, aggregation state, ligand stability, hydrocarbon uptake, and biodegradation efficiency under low-temperature conditions. Only after such validation can the computationally prioritized interfaces be translated into stronger claims about nano-assisted cold bioremediation.

## 5. Conclusions

This study presents an uncertainty-aware low-temperature xTB–MD–DFT screening workflow for prioritizing functionalized TiO_2_, ZnO, and CeO_2_ finite oxide surface-patch models according to hydrocarbon association and microbial-proxy perturbation descriptors. The revised analysis supports interfacial selectivity as the main design principle: candidate interfaces should not simply maximize hydrocarbon affinity, but should balance hydrocarbon association with low or moderate perturbation of microbial-envelope-related proxy targets.

Within the evaluated candidate library, TiO_2_–catechol and TiO_2_–glycerol remain the primary experimentally testable candidates, but they should be interpreted cautiously. TiO_2_–catechol is retained as an interface-conservative hypothesis because its original microbial-proxy interaction profile was mild and chemically interpretable. TiO_2_–glycerol is retained as an adsorption-oriented hypothesis because it combines hydrocarbon-association relevance with moderate perturbation against the peptide proxy in the completed short xTB-MD layer. However, TiO_2_–glycerol also showed very high displacement against the NAG proxy, and both TiO_2_-centered candidates carry computational uncertainty flags. They should therefore be interpreted as experimental hypotheses, not as validated biocompatible nanomaterials.

The sensitivity analysis broadens the candidate landscape. ZnO–catechol and ZnO–glycerol emerged as sensitivity-competitive secondary candidates under several scoring assumptions, indicating that the final interpretation should not be reduced to a rigid two-candidate ranking. In contrast, several CeO_2_-based systems, TiO_2_–citric acid, ZnO–citric acid, and other high-magnitude cases are better interpreted as warning or contrast interfaces because they displayed extreme descriptor magnitudes, strong target engagement, low-confidence MD behavior, unresolved refinement, or high proxy perturbation.

Methodologically, the work demonstrates how relative xTB descriptors, clean GFN2-xTB/ALPB rescoring, Tier-1/Tier-2 recovery, ORCA refinement, MD frame-quality auditing, completed short xTB-MD perturbation probes, sensitivity analysis, and final evidence classification can be combined into a transparent prioritization framework. The workflow does not provide experimentally calibrated adsorption-free energies, full nanoparticle behavior, explicit-solvent equilibrium dynamics, or direct microbial-toxicity evidence. Its value lies in reducing a broad oxide–ligand design space into a smaller and better-justified set of experimentally testable interfaces.

Future work should validate TiO_2_–catechol, TiO_2_–glycerol, ZnO–catechol, and ZnO–glycerol in psychrotolerant hydrocarbon-degrading systems through measurements of hydrocarbon uptake, bacterial growth, membrane integrity, aggregation state, ligand stability, surface charge, and biodegradation efficiency under low-temperature conditions. Longer explicit-solvent simulations, larger and hydroxylated surface models, pH- and ionic-strength-dependent charge regulation, competitive adsorption, dissolved organic matter, protein-corona effects, and full bacterial-envelope models would further strengthen the mechanistic basis of the proposed prioritization framework.

## Figures and Tables

**Table 1:** Screening design space and computational workflow inputs.

Candidate ID	Oxide Family	Surface Ligand	Multiplicity	n Atoms
CeO_2__patch__bare	CeO_2_	bare	1	12
CeO_2__patch__catechol	CeO_2_	catechol	1	26
CeO_2__patch__citric_acid	CeO_2_	citric acid	1	33
CeO_2__patch__glycerol	CeO_2_	glycerol	1	26
TiO_2__patch__bare	TiO_2_	bare	1	12
TiO_2__patch__catechol	TiO_2_	catechol	1	26
TiO_2__patch__citric_acid	TiO_2_	citric acid	1	33
TiO_2__patch__glycerol	TiO_2_	glycerol	1	26
zno_patch__bare	ZnO	bare	1	8
zno_patch__catechol	ZnO	catechol	1	22
zno_patch__citric_acid	ZnO	citric acid	1	29
zno_patch__glycerol	ZnO	glycerol	1	22

**Table 2:** Best hydrocarbon-associated configurations per candidate from xTB screening. The reported values are relative interaction-energy descriptors used for candidate prioritization, not experimentally calibrated adsorption free energies.

Candidate ID	Best Hydrocarbon	Best Pose	Best xTB Relative Interaction-Energy Descriptor (kcal/mol)	Complex ID
CeO_2__patch__bare	naphthalene	1	−3644.29	CeO_2__patch__bare__naphthalene__pose1
CeO_2__patch__catechol	toluene	3	−3942.98	CeO_2__patch__catechol__toluene__pose3
CeO_2__patch__citric_acid	naphthalene	2	−354.49	CeO_2__patch__citric_acid__naphthalene__pose2
CeO_2__patch__glycerol	naphthalene	1	−4108.94	CeO_2__patch__glycerol__naphthalene__pose1
TiO_2__patch__bare	toluene	3	4757.39	TiO_2__patch__bare__toluene__pose3
TiO_2__patch__catechol	naphthalene	1	−158.89	TiO_2__patch__catechol__naphthalene__pose1
TiO_2__patch__citric_acid	naphthalene	2	−4979.20	TiO_2__patch__citric_acid__naphthalene__pose2
TiO_2__patch__glycerol	toluene	1	−151.64	TiO_2__patch__glycerol__toluene__pose1
zno_patch__bare	naphthalene	2	−2424.03	zno_patch__bare__naphthalene__pose2
zno_patch__catechol	naphthalene	1	−147.87	zno_patch__catechol__naphthalene__pose1
zno_patch__citric_acid	hexane	2	−5339.88	zno_patch__citric_acid__hexane__pose2
zno_patch__glycerol	naphthalene	2	−165.13	zno_patch__glycerol__naphthalene__pose2

**Table 3:** Worst microbial-proxy interaction descriptors per candidate in xTB screening. The qualitative labels indicate relative target-engagement intensity within the reduced surface-patch model and should not be interpreted as direct toxicity classifications.

Candidate ID	Worst Target ID	Worst xTB Target Binding Energy (kcal/mol)	Complex ID	Interpretation
CeO_2__patch__bare	nag_proxy	−5266.80	CeO_2__patch__bare__nag_proxy__pose1	high target-engagement
CeO_2__patch__catechol	nag_proxy	−6124.31	CeO_2__patch__catechol__nag_proxy__pose3	high target-engagement
CeO_2__patch__citric_acid	surface_peptide_demo	−445.44	CeO_2__patch__citric_acid__surface_peptide_demo__pose1	high target-engagement
CeO_2__patch__glycerol	nag_proxy	−6706.25	CeO_2__patch__glycerol__nag_proxy__pose2	high target-engagement
TiO_2__patch__bare	surface_peptide_demo	−2412.15	TiO_2__patch__bare__surface_peptide_demo__pose1	high target-engagement
TiO_2__patch__catechol	surface_peptide_demo	−9.77	TiO_2__patch__catechol__surface_peptide_demo__pose2	mild
TiO_2__patch__citric_acid	nag_proxy	−197.21	TiO_2__patch__citric_acid__nag_proxy__pose1	moderate
TiO_2__patch__glycerol	nag_proxy	−16.71	TiO_2__patch__glycerol__nag_proxy__pose3	mild
zno_patch__bare	surface_peptide_demo	−7782.18	zno_patch__bare__surface_peptide_demo__pose1	high target-engagement
zno_patch__catechol	surface_peptide_demo	−42.03	zno_patch__catechol__surface_peptide_demo__pose2	mild-to-moderate
zno_patch__citric_acid	nag_proxy	−445.96	zno_patch__citric_acid__nag_proxy__pose2	high target-engagement
zno_patch__glycerol	nag_proxy	−101.71	zno_patch__glycerol__nag_proxy__pose1	moderate

**Table 4:** Completed short xTB-MD perturbation trajectories obtained from early frames of partial stable trajectories. The values are short-timescale perturbation descriptors and should not be interpreted as equilibrium MD, explicit-solvent sampling, toxicity evidence, or direct microbial compatibility evidence.

Candidate	Target Proxy	Completion Profile	Time (ps)	Temp. (K)	Frames	Mean RMSD (Å)	Max RMSD (Å)	Contact Persistence	Interpretation
TiO_2_–glycerol	NAG proxy	GFN2 gas	1.0	277.15	50	63.50	65.29	0.00	Very high proxy displacement
TiO_2_–glycerol	Peptide proxy	GFN2 gas	1.0	277.15	50	2.73	2.80	0.00	Moderate proxy perturbation
ZnO–catechol	Peptide proxy	GFN2 gas	0.5	235.00	50	53.63	54.53	0.00	Very high proxy displacement

**Table 5:** Final integrated ranking and decision summary.

Rank	Candidate ID	Hydrocarbon Binding Used (kcal/mol)	Target Binding Used (kcal/mol)	Target RMSD (Å)	Composite Score	Pareto-optimal	Decision Label
1	TiO_2__patch__glycerol	−37.51	−16.71	5.28	0.5373	True	lead adsorption-balanced
2	TiO_2__patch__catechol	−0.17	−4.14	1.88	0.5366	True	lead biocompatibility-balanced
3	zno_patch__citric_acid	−65.28	−445.96	3.51	0.5079	True	contrast case
4	zno_patch__catechol	−147.87	−42.03	53.28	0.4994	True	secondary contrast
5	CeO_2__patch__catechol	−3942.98	−6124.31	2.45	0.4832	True	caution
6	zno_patch__glycerol	−165.13	−101.71	70.95	0.4785	True	caution
7	CeO_2__patch__bare	−3644.29	−5266.80	49.06	0.4719	True	caution
8	CeO_2__patch__glycerol	−4108.94	−6706.25	2.41	0.4545	True	caution
9	TiO_2__patch__citric_acid	34.34	1204.99	1.74	0.4366	False	discard
10	CeO_2__patch__citric_acid	−354.49	−445.44	107.76	0.4343	True	caution
11	zno_patch__bare	−2424.03	−7782.18	18.80	0.1600	False	discard
12	TiO_2__patch__bare	4757.39	−2412.15	2.13	−0.1950	False	discard

## Data Availability

The data supporting the reported screening results are available from the corresponding author upon reasonable request.

## Abbreviations

The following abbreviations are used in this manuscript:

Abbreviation	Full term
ALPB	Analytical Linearized Poisson–Boltzmann
APC	Article Processing Charge
CeO_2_	Cerium dioxide
CPCM	Conductor-like Polarizable Continuum Model
DFT	Density Functional Theory
ETKDGv3	Experimental-Torsion Knowledge Distance Geometry, version 3
FASTA	FAST-All sequence format
GFN2-xTB	Geometry, Frequency, Noncovalent, extended tight-binding method, second generation
LPS	Lipopolysaccharide
MD	Molecular Dynamics
NVT	Constant number of particles, volume, and temperature ensemble
ORCA	ORCA quantum chemistry software package
PDB	Protein Data Bank
RDKit	Open-source cheminformatics toolkit
RMSD	Root-Mean-Square Deviation
SCF	Self-Consistent Field
SMILES	Simplified Molecular Input Line Entry System
TiO_2_	Titanium dioxide
UFF	Universal Force Field
xTB	Extended tight-binding
ZnO	Zinc oxide
